# Adequately assessing dehydration: A holy grail of paediatric emergency medicine

**DOI:** 10.1186/1865-1380-4-71

**Published:** 2011-11-24

**Authors:** Damian Roland, Colin Clarke, Meredith Borland, Elaine Pascoe

**Affiliations:** 1 Paediatric Emergency Medicine Leicester Academic (PEMLA) Group, Emergency Department Secretaries c/o Elizabeth Cadman-Moore, Leicester Royal Infirmary, Leicester, LE1 5WW, UK; 2 Emergency Department, Royal Perth Hospital, Perth Western Australia; 3 Princess Margaret Hospital for Children, Perth, Western Australia; 4 Division of Clinical Research and Education, Princess Margaret Hospital for Children, Perth, Western Australia; 5 School of Paediatrics and Child Health, University of Western Australia, Perth, Western Australia

## 

We read the work by Pringle at al. [1] with interest. One of the holy grails of Paediatric Emergency Medicine has been the rapid and reliable identification of the child with serious dehydration, and the converse, the ability to know when to safely discharge a child with a history of gastroenteritis. Recently there has been an external validation of a previously derived clinical dehydration scale by Bailey et al. [2]. It is encouraging to see this type of study as too often scoring systems are created without further testing. However we wondered about the generalisability of this result to routine Pediatric Emergency Care. Specifically we noted that in that study participating nurses undertook an additional training programme prior to study commencement. Is the score still valid if used by Pediatric Emergency Care staff who have not had this additional training? Our previous work has shown that experience and training in assessment may be vital in correctly assigning dehydration categories in children [3]. We found significant variability between junior doctors' assessments of dehydration compared to their seniors. We concluded previous studies on dehydration scoring systems may have benefited from well-trained staff and the introduction of these systems to naive health care professionals may not replicate initial results. The Pringle et al. study, while containing only a small number of subjects, challenges this conclusion again as it appears the care setting may influence the utility of the tool. The holy grail has yet to be found!

## References

[B1] Pringle Comparing the accuracy of the three popular clinical dehydration scales in children with diarrhea Int J Emerg Med 2011 4 58 10.1186/1865-1380-4-58 21902838 PMC3182880

[B2] Bailey B Gravel J Goldman R Friedman J Parkin P External validation of the clinical dehydration scale for children with acute gastroenteritis AcadEmerg Med 2010 17 583 588 10.1111/j.1553-2712.2010.00767.x 20624137

[B3] Roland D Clarke C Borland M Pascoe E Does a standardised scoring system of clinical signs reduce variability between doctors' assessments of the potentially dehydrated child? J Paediatr Child Health 2010 46 3 103 107 10.1111/j.1440-1754.2009.01646.x 20105256

